# What Are the Remedies?

**Published:** 1891-10

**Authors:** Robert Farley

**Affiliations:** Phœnixville, Pa.


### WHAT ARE THE REMEDIES?

ROBERT FARLEY, M. D., PHŒNIXVILLE, PA.

What remedy or remedies have

Sensation as if the whole body would pass away with the stool?

Sensation as if brain revolved?

Sensation as if stomach were scalded?

Sensation of swallowing over a lump in throat?

Sensation of something alive in abdomen?

Sensation of something alive in stomach?

Sensation of feet in an ant-hill?

Cephalalgia descending from vertex or occiput down the sterno-cleido-mastoid muscles?

Sweat of feet, profuse, stinking, corrosive, destroying stockings and shoes?

Chill begins in neck of bladder at end of the act of urination and spreads over entire body?

Can urinate only when standing?

Uterus feels swollen, as if dropsical?

Gurgling of air from urethra while urinating?

